# Characterization of CaCO_3_ Filled Poly(lactic) Acid and Bio Polyethylene Materials for Building Applications

**DOI:** 10.3390/polym13193323

**Published:** 2021-09-28

**Authors:** Ferran Serra-Parareda, Jesús Alba, Quim Tarrés, Francesc X. Espinach, Pere Mutjé, Marc Delgado-Aguilar

**Affiliations:** 1 LEPAMAP-PRODIS Research Group, University of Girona, C/Maria Aurèlia Capmany, 61, 17003 Girona, Spain; ferran.serrap@udg.edu (F.S.-P.); francisco.espinach@udg.edu (F.X.E.); pere.mutje@udg.edu (P.M.); m.delgado@udg.edu (M.D.-A.); 2 Centro de Tecnologías Físicas, Universitat Politècnica de València, EPS Gandia, C/Paranimf, 1, Grao de Gandia, 46730 Valencia, Spain; jesalba@fis.upv.es

**Keywords:** acoustic performance, gypsum, calcium carbonate, PLA, BioPE, mechanical properties

## Abstract

Noise pollution has been identified as a cause of a broad spectrum of diseases, motivating researchers to identify building materials capable of attenuating this pollution. The most common solution is the use of gypsum boards, which show a good response for low frequencies but have a poorer response for high frequencies. In addition, due to environmental concerns associated with buildings, the use of materials that minimize environmental impacts must be favored. In this research, two biopolymers, a poly(lactic) acid and a bio-polyethylene, were filled with two typologies of calcium carbonate, and their soundproofing properties were tested using impedance tubes. In addition, the morphology of the fillers was characterized, and here we discuss its impact on the mechanical properties of the composites. The results showed that the incorporation of calcium carbonate into bio-based thermoplastic materials can represent a strong alternative to gypsum, because their mechanical properties and sound barrier performance are superior. In addition, the inclusion of mineral fillers in thermoplastic materials has a positive impact on production costs, in addition to preserving the advantages of thermoplastics in terms of processing and recycling. Although the use of carbonate calcium decreases the mechanical properties of the materials, it enables the production of materials with insulation that is four-fold higher than that of gypsum. This demonstrates the potential of these materials as building lightweight solutions.

## 1. Introduction

Oil-based polymers have shown their value in a multitude of industrial sectors, such as automotive, construction and building, or product design. Such materials can be easily transformed into ready-to-use components at an affordable cost, while adding properties such as shape, color, or texture, which require a variety of processes for other materials. However, these polymers usually depend on a non-renewable resource and, due to growing environmental concerns, their use is becoming restricted. Nonetheless, the industrial use of plastics has continued to grow, and bio-based polymers have started to replace oil-based plastics in some uses [1,2,3,4].

Among bio-based polymers, poly(lactic acid) (PLA) has attracted significant attention of researchers [5,6]. This polymer is renewable and biodegradable, and, mainly due to its mechanical properties, is foreseen as a potential alternative to commodities and even some technical polymers [7]. In addition, PLA is a biodegradable polymer that exhibits a relatively high glass transition temperature and low melting temperature, whereas other commodity polymers such as polypropylene (PP) or polystyrene (PS) exhibit a higher melting point, which requires higher processing energy [8,9]. At present, the main uses of PLA encompass films and rigid structures for packaging, and particularly food packaging. However, PLA presents limitations in terms of gas barrier properties and low toughness, which excludes this material when moderate deformations or impact are foreseeable [10,11,12]. Several efforts have been undertaken to decrease this brittleness, mainly using blending with other polymers or copolymerization strategies [13,14].

Bio-polyethylene (BioPE) possesses the same chemical composition as oil-based polyethylene but originates from renewable sources. This material has industry-ready properties and can be reinforced and filled with the same fibers or fillers as the oil-based polyolefin. Compared to oil-based polyolefin, its main disadvantage is its cost. Currently, it is used for flexible packaging and films. Unlike PLA, BioPE has sufficient flexibility to deform without breaking, but lesser tensile strength and Young’s modulus. In addition, although being bio-based, it is not biodegradable [15,16]. The properties of PE filled with CaCO_3_ have been widely discussed in the literature [17,18,19].

One strategy to tailor the properties of a polymer is formulating a composite based on such a polymer. If a strong fiber–matrix interface is obtained, adding fibers increases the tensile strength and Young’s modulus of a material [20,21]. Typically, adding a cheap filler weakens the mechanical properties, while simultaneously reducing its cost. The main disadvantage of the use of PLA and BioPE as construction materials are their cost compared to commodity materials such as gypsum. To reduce these costs, materials with low value, usually powders, are mixed with polymer matrices. Calcium carbonate (CaCO_3_) is one of the most widely used fillers for polymers [22,23,24], and can be added in high loadings. Thus, if the changes in the mechanical properties of the material do not disqualify its use for some applications, cost reductions can be beneficial. Moreover, due to other properties, such as soundproofing and environmental impact, the resulting materials can be profitable [25].

It is known that fillers influence the soundproof abilities of composites. The literature shows the addition of natural fillers, such as stone groundwood, waste orange pruning fibers, or olive stones, can increase the ability of polypropylene to absorb some sound frequencies [26,27,28,29]. Sound absorption depends on the intrinsic properties of the materials, the size, cross-section, and dispersion of the particles, and the density, thickness, and porosity of the composite material [29]. Sound waves can be transmitted through the material, or can be absorbed or reflected. The ability to absorb the waves that are not reflected by the material is measured as the sound absorption properties of a material. Sound waves propagate through the material as sound energy that can be transformed into kinetic energy, and subsequently into heat, due to vibrations and intermolecular friction [30]. These soundproofing properties are of special interest when materials are used in the construction field. In part, this is because noise pollution has been identified as the cause of health problems such as hypertension, and psychological symptoms such as anxiety [31,32,33]. Thus, addressing the problem of sound pollution in buildings involves the elimination of sound or its reduction to acceptable levels. One of the most common solutions in architecture involves the use of lightweight construction materials such as gypsum boards. Compared to masonry, gypsum boards have the advantage of being faster to build as a form of dry construction, and having less weight per area unit. Nonetheless, frequencies around 2500 Hz, which include music or talking, are poorly masked by gypsum boards [34]. Thus, the use of other materials that can increase the sound insulation at the cited frequencies can be of interest for architects and engineers [26,27,35,36,37,38,39]. The construction and building industry is continuously in the search of innovative solutions, particularly those that are more lightweight, sustainable, and cost-efficient [40,41,42].

Moreover, the European Union has developed regulations to ensure the quality of the soundproofing levels of buildings and installations. Although some of these regulations are guidelines, others are mandatory, and it is possible that the number of mandatory regulations will increase in the future. These regulations include limit values for airborne and sound insulation, acoustic absorption, reverberation time, and traffic noise. For example, DS 490:2007 proposes classes from A to F to evaluate dwellings; to obtain an A grade, airborne insulation must be higher than 68 dB [43]. The foreseeable evolution of these regulations in the EU and other regions increases the importance of innovative solutions to noise pollution. Composites based on hybrid fiber reinforcements, manufactured from high-volume and problematic local post-consumer wastes, such as textiles, mattresses foams, and mixed wood, have been proven to be able to absorb high-frequency sounds (>2000 Hz), for which gypsum boards show a decrease in soundproofing properties [44]. Other researchers have examined the use of waste material, such as rubber tire crumbs and wooden scrap present in the environment, thus obtaining materials that can be used in building applications, acoustic panels, and acoustic enclosures [45]. Nevertheless, previous research has exposed existing problems, such as the behavior of the materials under fire conditions. The literature also shows a high number of studies devoted to the use of lignocellulosic fibers as polymer filler, to obtain materials with enhanced soundproof properties [46,47]. The use of natural fibers increases the response for frequencies higher than 2000 Hz, at which gypsum boards tend to perform poorly. Other researchers have used inorganic fillers such as calcium carbonate to prepare composite materials with enhanced soundproofing capabilities [48,49]. The researchers found that the insulation properties of the matrix were improved significantly, but nonlinearly, with the presence of nano-CaCO_3_. These studies show the value of calcium carbonate as filler to enhance the soundproofing properties of composites.

The objective of this research was to use calcium carbonate as filler in two bio-based polymers (PLA and BioPE) to characterize sustainable and environmentally friendly materials to be used for building purposes. Mechanical properties were examined, including via tensile, flexural, and impact tests, and are discussed herein. The impact of the percentage of calcium carbonate on these properties, and the effect of the nature of the filler, shows the ability of the composites to be used instead of gypsum boards. It was found that the morphology and granulometry of the fillers played a role in the mechanical properties. One of the main disadvantages of gypsum boards as acoustic insulation materials is their poor response against sound frequencies between 2000 and 5000 Hz. The use of calcium carbonate shows a noticeable increase in sound transmission loss.

CaCO_3_-filled materials are expected to deliver better sound insulation properties than gypsum boards, especially at high frequencies. This increases its potential for application in cases where such high frequencies are expected, both for interior or exterior sound insulation.

## 2. Materials and Methods

### 2.1. Materials

The selected matrices were polylactic acid (PLA) Ingeo^TM^ Biopolymer 4043D from NatureWorks LLC, which was provided by Resinex Spain S.L. (Vilallonga del Camp, Spain), and bio-based high-density polyethylene (BioPE) I’m green^TM^ SHA7260, supplied by Braskem (São Paulo, Brazil). Regarding mineral fillers, ground calcium carbonate (GCC) was kindly provided by Cales de Llierca S.A. (Argelaguer, Spain), and precipitated calcium carbonate (PCC) was kindly supplied by Omya AG (Oftringen, Switzerland).

### 2.2. Chemical, Physical and Morphological Analysis of GCC and PCC

The morphology of both GCC and PCC was determined using laser diffraction analysis in a Mastersizer 2000 from Malvern Instruments Ltd. equipped with the software Mastersizer 2000 v5.60. The equipment can identify particles with sizes ranging from 0.02 to 2000 µm and returns the granulometry of the analyzed particles in the form of a diameter distribution in volume.

Both GCC and PC were also observed using field emission scanning electron microscopy (FE-SEM). Samples were placed on the top of an adhesive tape and subjected to a nitrogen flow to remove the non-adhered particles, to be directly observed in a HITACHI S-4100 microscope operating at an accelerating voltage of 7 kV. Energy-dispersive X-ray (EDX) spectroscopy was also performed, obtaining an elemental analysis of the mineral fillers. EDX was performed considering only carbon, oxygen, and calcium, in one analysis, and considering all the elements that the equipment detects, in another.

Density was determined using a pycnometer.

### 2.3. Composite Preparation and Test Specimen Obtaining

GCC and PCC were mixed with PLA and BioPE at different weight ratios using an intensive kinetic Gelimat mixer. The processing conditions were the same for GCC and PCC, and for PLA and BioPE, with the exception of the melting temperature, which was automatically adjusted to the melting point of each matrix. First, the polymer matrix was introduced into the mixer drum at 300 rpm. Then, the mineral filler was incorporated into the drum at the same rotational speed. Finally, the mixing process was conducted for 2 min at 2350 rpm until achieving the melting point of each matrix to be discharged. PLA composites were discharged at 200 °C and BioPE composites were discharged at 190 °C.

The resulting composites were then milled using a knife mill, to be later processed in an Allrounder-220 M injection molder from Arburg (Eschweiler, Germany), obtaining ASTM D638 standard specimens. The temperature profile for injecting PLA-based composites was 175/180/185/190/195 °C from the first to the fifth temperature region, respectively, and the cooling time in the mold was set at 30 s. The injection pressure was varied from 250 to 375 bar, corresponding to the neat matrix and the most charged material, respectively. In the case of BioPE-based composites, the temperature profile was set at 160/165/170/175/180 °C, and cooling time at 20 s. In this case, the injection pressure was varied from 275 to 450 bar.

Hereafter, the materials are referred to by the acronym of the matrix (PLA or BioPE), followed by the percentage of GCC or PCC. As an example, composites prepared from BioPE and 40 wt% of PCC are labeled BioPE/40PCC.

### 2.4. Evaluation of the Mechanical Properties

**Figure 1:**
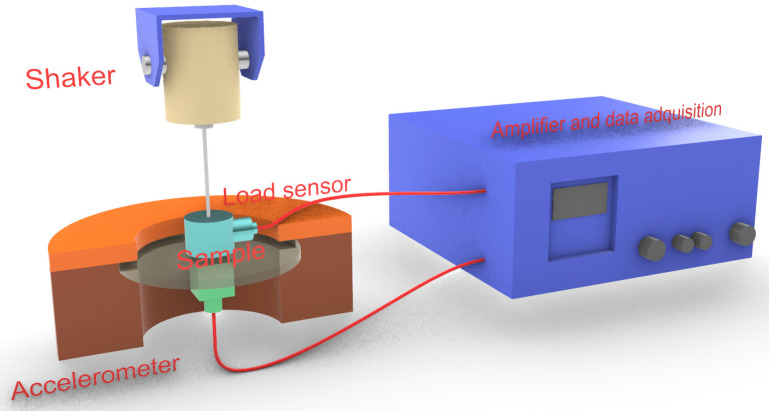
Mechanic resonance equipment.

Tensile properties were evaluated using two pieces of equipment. A mechanic resonance apparatus designed at the University Polytechnique of Valencia (Figure 1) was used to determine Young’s modulus, density, surface density, and loss factor. This device is based on the literature and is composed of a TIRA S 50009 and TIRA BAA 60 shaker and amplifier, respectively. The shaker transmits vibrations to a sample positioned over an ENDEVCO Isotron^®^ 2311-100 load sensor, able to measure from −220 to 220 N. The device also uses a Brüel&Kjaer 4508-B-001 accelerometer. All the information from the accelerometer and the load sensor is sent to a measure acquisition system and used to compute the mechanical properties using a MATLAB^®^ routine.

The Young’s modulus (E, GPa) of the fired samples was measured by the impulse excitation of the vibration technique (Sonelastic, ATCP, Ribeirão Preto, Brazil), according to the ASTM E 1876-01 standard (“Standard Test Method for Dynamic Young’s Modulus, Shear Modulus, and Poisson’s Ratio by Impulse Excitation of Vibration”).

Tensile strength, Young’s modulus, and strain at break were also evaluated with a DTC-10 Universal testing machine by IDMtest, following ASTM D790. Dog-bone specimens with measurements of approximately 160 × 13.3 × 3.2 mm measures were placed in a conditioning chamber at 23 °C and 50% relative humidity for 48 h before testing, following ASTM D618. This procedure was also followed for flexural and impact specimens. The test equipment fits a 5 kN load cell. Reported values are the average of at least 5 samples. Strains to measure Young’s moduli were determined with an extensometer.

To measure flexural properties, specimens according to ASTM D618 were tested under three-point bending. The equipment was the same as that used for tensile testing. Reported results are the average of at least 5 samples. The flexural strain at break ($\varepsilon_{f}^{C}$) was computed from Equation (1):

(1) $$\varepsilon_{f}^{C}=\frac{6\cdot D\cdot d}{L^{2}}$$

where *D* is the maximum deflection at the center of the specimen, *d* is the specimen depth, and *L* is the support span, which is 52.6 mm in the present experiment.

Notched and unnotched specimens were used to test Charpy impact strength following ISO 179 specifications. The strengths were measured in a Resil 5.5 pendulum device by Ceast, which is able to measure from 0.5 to 5.5 Joule. Energy absorbed under the Izod impact test, following ISO 180, was also measured using the same equipment.

### 2.5. Acoustic Characterization

**Figure 2:**
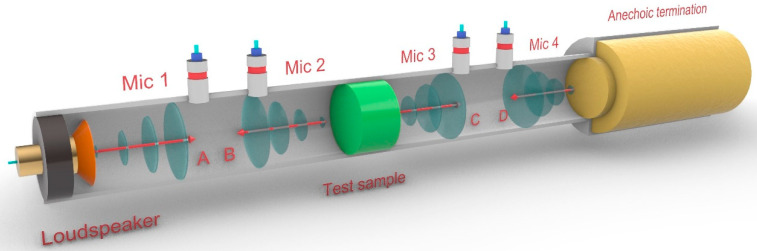
Experimental setup of the impedance tubes for acoustic characterization.

The literature shows multiple methods to evaluate acoustic insulation, of which some are based on impedance tubes [35,50]. Transmission loss (TL) was measured according to the ASTM E2611—19 standard (“Standard Test Method for Normal Incidence Determination of Porous Material Acoustical Properties Based on the Transfer Matrix Method”) using a method developed at the Polytechnic School of Gandia and based on the impedance tubes test. The method was developed to address a lack of procedures to measure transmission loss from data recovered from impedance tube-based equipment. Figure 2 shows a scheme of the apparatus.

The device incorporates two impedance tubes, one after the test sample and the other before, measuring 1315 and 1233 mm, respectively. The tubes have a 40 mm interior diameter. The tubes allow positioning two microphones at 3 different distances. These distances were designed to determine the frequency spectrum for evaluation, ensuring a plane propagation of the waves inside the tube. For the present research, the microphones were placed at a distance of 32 mm. A loudspeaker placed at the end of the first impedance tube is used to generate plane waves. Two of the microphones are placed between the loudspeaker and the sample, and the other two between the sample and an anechoic termination. The device symbolizes a transference matrix of the incident and reflected waves from both impedance tubes. If this matrix is known, then *TL* can be obtained from the following equation:

(2) $$TL=20\log_{10}\left|\frac{e^{jks}-H_{12}}{e^{jks}-H_{34}}\right|-20\log_{10}\left|H_{t}\right|$$

In the equation, *S* marks the distance between the microphones. *H_t_* is the relation between auto spectrums. *H*_12_ and *H*_34_ are the transference function between the first and the second, and the third and the fourth, microphones, respectively. These functions are defined by:

(3) $$H_{i,i+1}=P_{i+1}/P_{i}$$

*P_i_* is the complex acoustic pressure measured by microphone *i*.

*H_t_* is defined by:

(4) $$H_{t}=\sqrt{\left|S_{d}/S_{u}\right|}$$

where *S_u_* is the auto spectrum at the first impedance tube, and *S_d_* is the auto spectrum at the second tube, obtained from:

(5) $$S_{d}=P_{3}\cdot P_{4}^{*}$$

(6) $$S_{u}=P_{1}\cdot P_{2}^{*}$$

where *P*_2_^*^ and *P*_4_^*^ are the complex conjugates of the complex acoustic pressure measured by the 2nd and the 4th microphones.

Global insulation was obtained from the coupling effect between acoustic impedances of the samples. Indexes of sound reduction (R) for the different materials were obtained and represented against sound frequencies. A global value (RA) for all the materials was used to compare the average soundproofing capabilities of the materials.

### 2.6. Color Analysis of the Composites

Color information was acquired with a Canon PowerShot G12 digital camera. The samples were photographed under white light, inside a lightbox. The temperature of the light, measured with a Gossen Mastersix light meter, was 5200 K. The resulting archives were analyzed in Inkscape^®^ to evaluate the color by measuring a circular region with a diameter of 100 pixels in the central area of the samples. Information on the RGB and HSL coordinates of the colors was extracted.

## 3. Results and Discussion

### 3.1. Characterization of GCC and PCC

**Figure 3:**
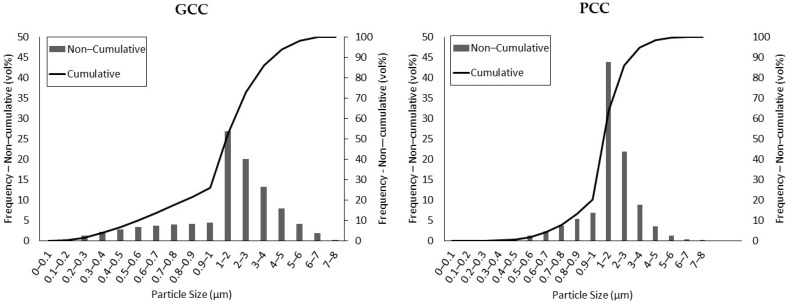
Cumulative and non-cumulative particle size distributions of GCC and PCC.

GCC and PCC showed average diameters of 1.885 and 1.627 µm, respectively. PCC exhibited a more regular distribution of diameters with an almost normal shape, whereas GCC showed an asymmetric distribution, with a higher presence of smaller particles (Figure 3).

In the case of GCC, 25.98% of the particles exhibited a size below 1 mm, whereas in the case of PCC, this percentage accounted for 20.09%. Although the differences were almost negligible, this implies that for a certain filler fraction in the composite, there will be more GCC particles than in the case of PCC. This should hinder the dispersion of GCC compared to PCC. Nonetheless, the presence of a higher number of particles can increase the sound barrier effect of CaCO_3_, because the wave transmission medium is more heterogeneous. By comparison, in the case of GCC, a higher number of particles per area unit can increase the probability of fracture initiation points. To the same extent, GCC powder exhibited a higher presence of larger particles than in the case of PCC, which may become detached from the matrix when the material is under load. Larger particles will create larger holes, and the probability of reaching a critical size and fracture propagation phenomena may increase.

**Figure 4:**
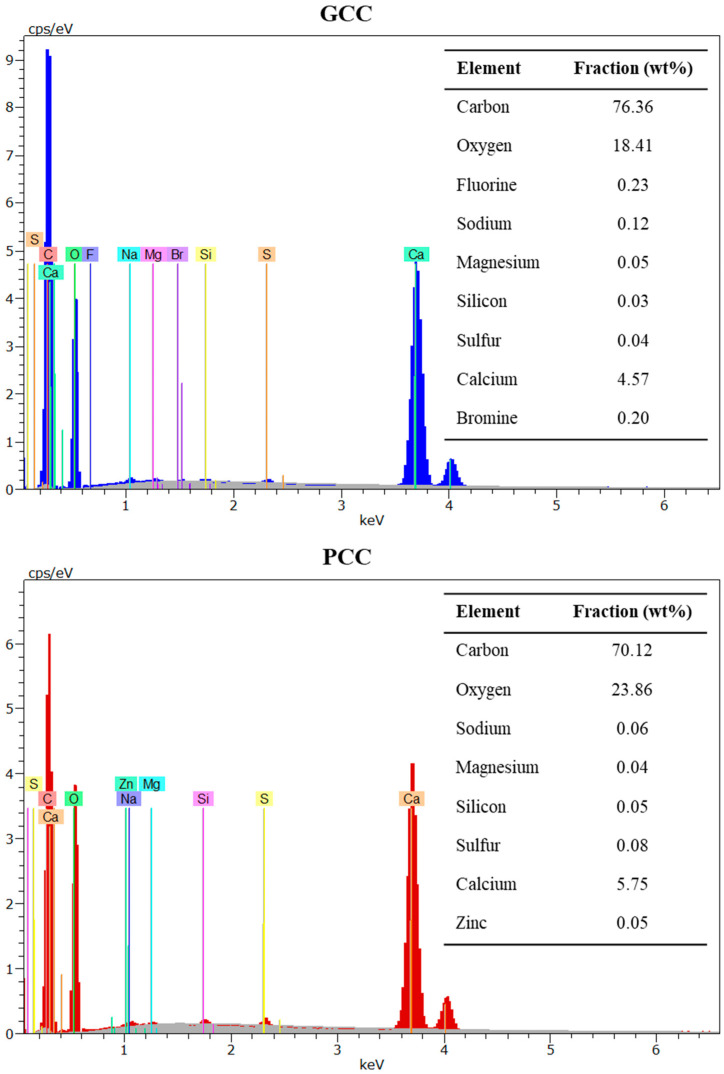
EDX of GCC and PCC.

In terms of density, no significant differences were observed, because the values were found to be 2686 and 2720 kg/m^3^ for GCC and PCC, respectively, which is in agreement with previously reported values [18,19]. Regarding chemical composition, GCC and PCC did not exhibit remarkable differences through EDX analysis, as the contents of carbon, oxygen, and calcium were very similar. Only a small number of differences were observed in the impurities, as an almost negligible trace of zinc was found in the PCC sample (Figure 4).

**Figure 5:**
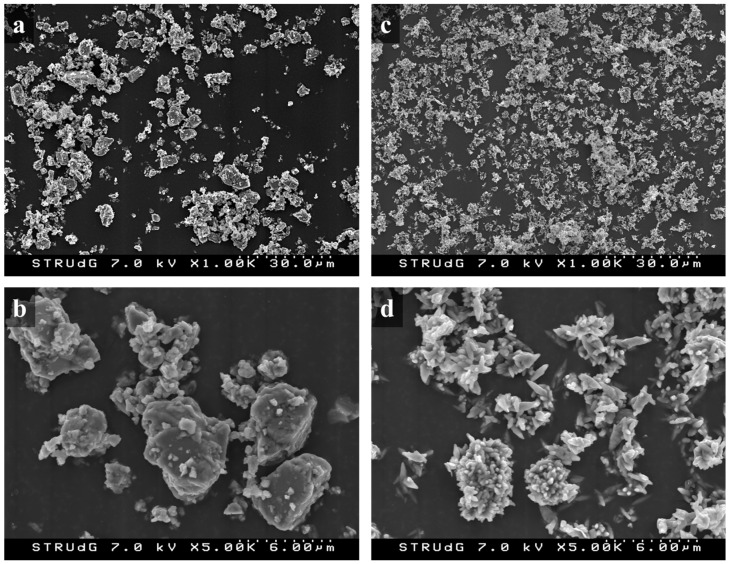
FE-SEM images of GCC at low (**a**) and high (**b**) magnification, and PCC at low (**c**) and high (**d**) magnification.

Morphology was also assessed using FE-SEM, as reflected in Figure 5. The obtained micrographs confirm the calculated average diameters through laser diffraction analysis, because GCC particles are slightly larger than those from PCC. In addition, the narrower distribution of PCC can also be qualitatively observed by comparing the FE-SEM images at low magnification.

**Figure 6:**
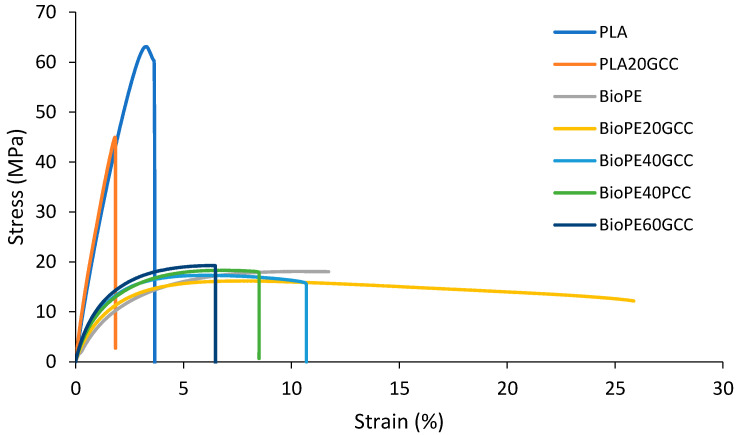
Stress–strain curves for the matrices and the composites. The curves belong to one of the tested specimens for any of the formulations.

GCC exhibited more rounded particles and softer surfaces than PCC, mainly due to the differences in the extraction process. Although GCC is directly extracted from the grinding process of limestone rock, PCC is chemically produced using quicklime (originating from limestone burning) carbonation, and finally precipitated. This precipitation process, together with the natural gas-driven calcination process at high temperatures (above 900 °C), ensures the high purity of the resulting CaCO_3_ particles. As reflected in Figure 4 and Figure 6, the size distribution of GCC is broader than in the case of PCC, which may negatively affect the resulting properties of CaCO_3_-filled thermoplastic materials. The surfaces of the GCC and PCC particles are also significantly different, as crystals are observed in the chemically obtained CaCO_3_. The presence of these crystals generates sharp surfaces that may be susceptible to generate concentrated loads, which may negatively affect the mechanical properties of the resulting PCC-filled composites [51,52]. In addition, the irregularities on the PCC surface may hinder the wettability of the filler by the molten matrix, which may result in air bubbles inside the composite and, thus, negatively affect the acoustic insulation of the material, as discussed below.

### 3.2. Physical Characterization of the Composites

The physical characterization of the obtained composites consisted of density measurements and color analysis. As expected, regardless of the matrix (PLA or BioPE) or the filler (GCC or PCC), density increased as the amount of filler was increased (Table 1).

Surprisingly, the composites filled with PCC exhibited a lower density than those prepared from GCC, although PCC exhibited a higher density than GCC. This may confirm the presence of voids or air bubbles within the composite due to the sharp surface of PCC, which may hinder the wettability of the filler. As expected, BioPE-based composites exhibited lower density than those prepared from PLA, as the density of the matrix is lower.

Table 2 shows the HSL (hue, saturation, and lightness) coordinates of the material colors, in addition to a color sample of the material.

The base color for PLA was an unsaturated yellow. The incorporation of GCC changed the hue towards a greenish tone, decreasing the saturation and luminance. Colors changed only slightly from one percentage of filler to another, and between natural or precipitate CaCO_3_-filled materials. PLA matrix was translucent, and lost this property when CaCO_3_ was added, becoming opaque. BioPE showed an unsaturated cyan with high luminance that, at first sight, looked white. When GCC was added to the material, the color changed to unsaturated yellow, with similar luminance. Adding PCC resulted in an unsaturated yellow color. The low saturation of the specimens resulted in losing almost all hue information, and the colors became shades of grey at first sight. Considering that the obtained composites may have potential application as construction panels, color must be considered as a limiting characteristic, because it may influence the resulting color of the wall once painted. Similar effects are found when a matrix is filled with natural fibers. In the case of polypropylene filled with fibers from orange tree pruning, composites acquired a brownish color as the amount of filler was increased [26].

### 3.3. Mechanical Characterization of the Composites

Gypsum boards are rarely under tensile loads when in use but, during their transportation and manipulation, boards may be subjected to tensile stresses. This material usually shows poor tensile properties, compared to any polymer, and poor ability to deform without breaking because of its brittleness. The use of materials prone to be manipulated under tensile loads can facilitate such manipulation and avoid material losses due to breaking.

Table 3 shows the mechanical properties of the materials obtained from their tensile test, with the exceptions of PLA40GCC and PLA40PCC, which were impossible to test due to their excessive brittleness. The table includes tensile strength (σ_t_), strain at maximum strength (ε_tM_), strain at break (ε_t_), and Young’s modulus (E_t_).

The selected matrices, PLA and BioPE, exhibited significantly different original tensile properties. The tensile strength of PLA was 62.58 MPa, which is in agreement with the literature, particularly for the 4043D grade of Natureworks, and significantly higher than some commercial polypropylene (PP), and the same magnitude as that of some grades of polyamide [2,7]. By comparison, BioPE exhibited similar properties to other commercial grades of high-density polyethylene (HDPE), both in terms of strength and modulus, and in terms of elongation at maximum stress. Elongation at break was not measured because it reached the limit of the testing equipment.

The inclusion of a 20 wt% of GCC in PLA decreased the tensile strength by 27.3%, whereas a 31.9% increase was found in Young’s modulus. This increase in stiffness was observed in the reduction in the elongation, both at break and maximum stress. Although the tensile strength was significantly decreased, that of the resulting material was 45.48 MPa, which is higher than that of some standard polyolefin, such as PP, and three-fold higher than that of BioPE [53]. The reduction in the tensile strength of the material due to the inclusion of mineral fillers has been previously reported, and can be attributed to a weak interface between the fillers and matrix. In addition, the fillers may act as stress concentration points, which may result in weaker structures with a lower capacity to withstand deformations [54]. Figure 6 shows the stress–strain curves of composites with the assayed formulations.

In the case of BioPE-based materials, the incorporation of a 20 wt% GCC reduced the tensile strength of the material by 10.9% compared with that of the matrix. Contrarily, a 40 wt% content of both GCC and PCC increased the tensile strength of the resulting materials, taking as reference the BioPE20GCC. Although the average values were lower than the strength shown by the matrix, an ANOVA at a 95% confidence rate shows that the tensile strengths of BioPE and the composites filled with 40 wt% of GCC or PCC cannot be considered different. Further increases in the percentage of filler showed an increase in the tensile strength, so that BioPE60GCC showed a 6% increase compared to the matrix. In statistical terms, both GCC and PCC can be considered to not influence the tensile strength of BioPE at 40% filler contents, as revealed by the ANOVA.

Some differences were found between the strain at break and the strain at maximum stress. These differences were more noticeable in the case of BioPE-based materials than in the case of PLA composites. This can be attributed to the higher ductility of BioPE compared to PLA, which exhibited a brittle behavior, with low differences between both strains. As expected, the strain at maximum strength and break of the materials decreased when a more brittle phase was added to the composite, namely GCC and PCC. At the same time, the differences between both strains decreased as filler content was increased. The strain at maximum strength and break of BioPE60GCC are statistically equal. The kind of filler or its percentage had no statistical impact on the strain at a maximum strength of BioPE-based materials with 40 and 60 wt% filler contents. By comparison, materials filled with GCC showed higher strains at break than those filled with PCC, and were statistically equivalent to BioPE40PCC and BioPE60GCC. The literature shows similar results when CaCO_3_ was added to a PE matrix [17]. The use of lignocellulosic particles with a low aspect ratio showed similar behaviors regarding tensile strength. Polypropylene filled with olive stone flour showed a slight trend to increase the tensile strength of the matrix, but at high fill percentages, this strength tends to decrease [55]. Nonetheless, in the case of lignocellulosic fillers, the use of coupling agents can increase the chemical interactions between the filler surface and the matrix, obtaining materials with stronger interfaces and thus higher tensile strengths. Regardless, however, this means using more reactants and thus does not agree with the principles of green chemistry [56].

Young’s modulus of the materials increased with the addition of filler. This was expected due to the highest rigidity of CaCO_3_, compared to the selected matrices. In the case of PLA-based materials, PLA20GCC showed a Young’s modulus that was 31.9% higher than that of PLA, and significantly higher than that of a standard polyolefin [57,58]. In the case of BioPE-based materials, Young’s modulus increased linearly with the percentage of filler. The linear evolution of Young’s modulus has been reported to be indicative of good dispersion of the filler, regardless of its nature, morphology, or compatibility with the matrix [59,60,61,62].

PLA20GCC showed a more brittle nature than any BioPE-based materials. Young’s modulus and tensile strength of this material were higher, and its strain at break noticeably lower, than those of any BioPE-based material. BioPE-based materials showed a higher ability to deform without breaking. All of the obtained materials, even the neat matrices, exhibited higher tensile strength than that of gypsum board, which is typically used for interior panels and has a tensile strength of 3.58 MPa [63].

The literature proposes an air hole mechanism for the failure of materials filled with CaCO_3_. This mechanism is based on the weak interface between the matrix and filler particles. The particles are easily debonded from the matrix, creating voids or air holes. As the specimens are stretched, the air holes grow and can also merge [17]. Furthermore, the weak interface inhibits load transfer from the matrix to the fillers, decreasing the resistant section of the matrix, and decreasing its toughness [54].

CaCO_3_ and olive stone flour-filled materials showed similar behaviors of their Young’s moduli. The inclusion of a stiffer phase in the composite, regardless of its nature, increased the rigidity of the composites and decreased their ability to deform without breaking [55].

**Figure 7:**
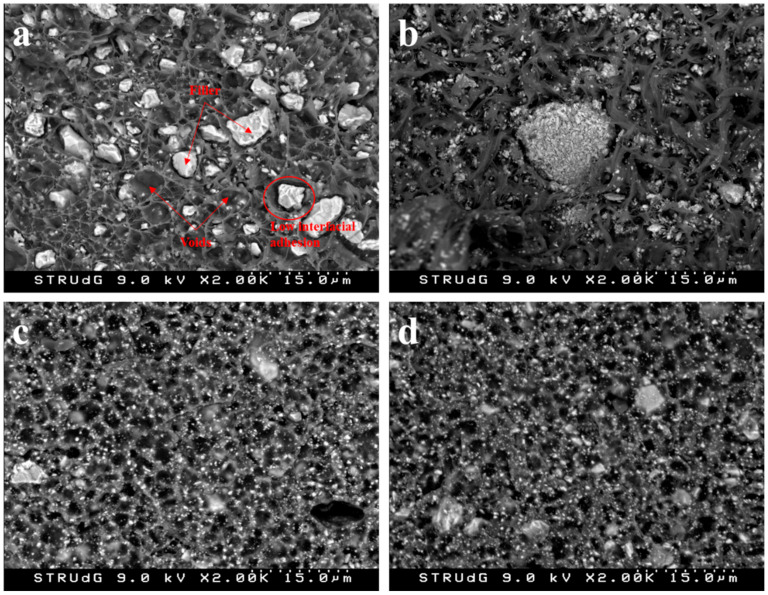
FE-SEM images of the fracture surface of BioPE/40GCC (**a**), BioPE/40PCC (**b**), PLA/40GCC (**c**), and PLA/40PCC (**d**).

The appropriate dispersion of both PCC and GCC in the selected matrices was confirmed by means of analyzing the fracture surface of the tested specimens by FE-SEM (Figure 7). In addition, some voids were identified, confirming the air hole mechanism, which may negatively influence the mechanical properties of the resulting composites.

**Figure 8:**
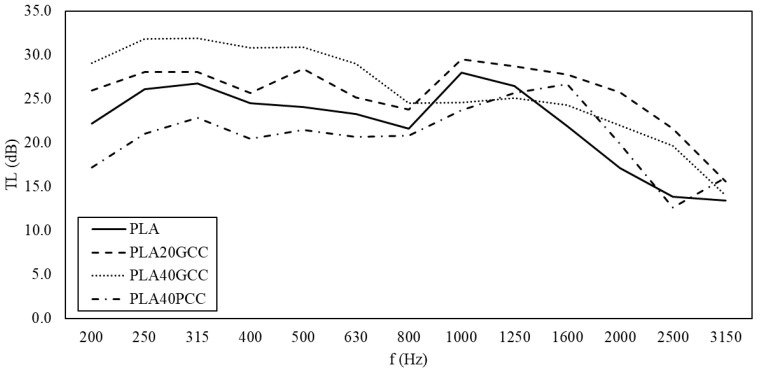
Transmission loss versus the frequency measured in the tested PLA-based composite materials.

Figure 8 reveals that fillers, both GCC and PCC, were properly dispersed within the BioPE and PLA matrices. However, after tensile testing, several voids can be observed at the surface of the fracture region, which clearly indicates a filler detachment from the matrix. Furthermore, the region surrounding the fillers appears to not by properly bonded, which suggests a low interfacial adhesion between GCC/PCC and BioPE or PLA. Thus, in addition to the low aspect ratio of the fillers, the low interfacial adhesion also contributed to the decrease in the tensile strength of the resulting composites.

Flexural loads are more common than tensile loads in the case of gypsum boards, particularly when their use is intended for interior panels in the building industry. Tensile properties of gypsum boards are rarely found, whereas flexural properties are extensively reported. At 20 °C, gypsum boards have been reported to show 1.61 MPa of flexural strength, 1.57 GPa of flexural modulus, and 0.1% strain at failure under flexural loads [64]. Table 4 shows the mechanical properties of the materials obtained from their three-point bend flexural test. The table includes flexural strength (σ_f_), the maximum deflection of the center of the specimen (D), strain at failure (ε_f_), and flexural modulus (E_f_).

As in the case of tensile properties, PLA-based materials showed higher properties than BioPE-based composites. In this case, it was possible to measure the flexural properties of the materials by adding 40 wt% of GCC. The flexural strength of the matrix was 67 and 459% higher than the materials adding 20 and 40 wt% of GCC, respectively. Moreover, the ability of the materials to deform without breaking decreased dramatically and, although the matrix withstood 6.9 % strains, PLA40GCC reduced this strain to 0.43% (ten times less). In the case of the flexural moduli, its value increased with the inclusion of GCC up to 21% higher than the matrix for 40 wt% filler contents. In terms of flexural strength and modulus, the values for the BioPE matrix and the PLA4GCC were statistically equivalent. Nonetheless, BioPE was able to bear higher deformations without breaking.

BioPE-based materials exhibited enhancements in the flexural strength and the flexural modulus with the percentage of filler. The flexural load in the section of the specimen is comprised such that half of the section is under tensile loads and the other half is under compressive loads. As gypsum, CaCO_3_ shows better compressive properties than tensile properties. Thus, the increase in the flexural properties compared to the tensile properties is due to this anisotropy. GCC and PCC returned equivalent flexural strengths and moduli. The Strain at break of BioPE-based materials was found to be lower than that of the matrix, but changed slightly with the percentage of filler. As in the case of the tensile strength, the strain at break of the materials filled with 40 wt% of PCC was statistically equivalent to that of the material filled with 60 wt% of GCC. Flexural moduli linearly increased with the percentages of filler and BioPE60GCC showed a 91% increase relative to the matrix. The flexural properties of the materials were higher than those of gypsum boards [64].

The differences between the tensile and flexural properties of a material are an indication of anisotropy. In the case of materials filled with rigid particles, the differences between both strengths are noticeable [65]. The results obtained with BioPE-filled CaCO_3_ are similar to those obtained for uncoupled lignocellulosic fiber-filled polyolefin [66].

Tensile and flexural properties are useful to predict the behavior of a material under constant loads. Nonetheless, construction materials can be subjected to impact over their lifetime. Thus, it is of importance to assess the ability of the materials to absorb impact energy. Table 5 shows the results of the impact strength test. The table includes the results of the unnotched and notched Charpy test and the Izod test.

As expected due to its brittleness, PLA exhibited lower impact strength than BioPE. Unnotched Charpy and Izod tests showed similar behaviors, with noticeable decreases when the filler was added to the composition of the material. This behavior was expected, because the presence of fillers within the matrix may generate points at which the fracture can propagate. In addition, assuming that the composites followed an air hole mechanism, there may be weak regions surrounding the fillers. The impact strength of the composites noticeably decreased with the amount of filler, because PLA20GCC and PLA40GCC materials exhibited 1.48- and 9.1-fold lower impact strengths than that of the neat matrix. By comparison, BioPE and its composites showed a higher ability to absorb energy. BioPE and BIOPE20GCC were able to absorb more than 5.5 J, the maximum impact load of the equipment. The materials at 40 and 60 wt% filler contents exhibited Charpy impact strengths lower than that of the matrix. GCC-based materials showed impact strengths that were higher than those of the PCC-based materials. This may be attributed to the morphology of the fillers. As explained above, the sharp surface of PCC may limit the interactions between matrix and filler, which may promote the generation of air holes in the PCC surroundings. The impact strength of BioPE40GCC was lower than that of the matrix and BioPE60GCC. As expected, the Izod test revealed similar outputs. To break an unnotched test under impact, the specimen must absorb the energy devoted to creating a fracture and then the energy devoted to its propagation. In the case of PLA-based materials, notched specimens showed decreasing impact strengths when the percentage of filler was increased. Nonetheless, the impact strengths of PLA and PLA20GCC were statistically equivalent, signaling the importance of the matrix in fracture propagation. Tensile results showed a poor adhesion between the filler and PLA. Thus, the fracture possibly propagates through the matrix and the interphase, detaching CaCO_3_ particles from the matrix.

In the case of BioPE-based materials, notched impact strength decreased for the materials with 20 and 40 wt% contents and then increased for the material with a 60 wt% content. The reasons for the decrease in the energy needed to propagate the fracture may be similar to those in the case of PLA-based composites, i.e., weak interphase between the particles and the matrix, the matrix as main propagation media, and the presence of air holes within the matrix. The slight increase observed for BioPE60N may be due to a more complex fracture propagation path due to the number of CaCO_3_ particles. These particles can divert the propagation path and require more energy for propagation.

BioPE-based composites and polypropylene filled with olive stone flour showed a similar evolution to their notched impact strength [55]. The literature shows the importance of the filler morphology compared to the impact strength of the composites, because elliptical shapes, such as those of GCC, can promote stress concentration in the tip of the particles. In terms of mechanical properties, short fiber-reinforced polymers have advantages compared to CaCO_3_-filled plastics, showing noticeable increases in the strength and stiffness of the composites with the percentage of reinforcement. In addition, the density of lignocellulosic fibers is lower than that of CaCO_3_, and thus, its composites are lighter. Thus, in terms of mechanical performance, the use of CaCO_3_ has a lower impact than that of natural fibers. Nevertheless, CaCO_3_ mechanical properties are comparable to those of gypsum, and, as discussed below, the soundproofing properties of these materials indicate its advantages as a building material.

### 3.4. Acoustic Properties

Figure 8 shows the transmission loss versus the frequency measured in the tested PLA-based composite materials. The test was performed in the impedance tube defined in the Materials and Methods section.

Regarding PLA, PLA20GCC showed an increase in sound insulation. The sound insulation ability of PLA40GCC increased for the low frequencies but did not for the high frequencies. Thus, in the case of PLA to which natural GCC was added, there was a measurable improvement in terms of acoustic insulation. In contrast, the use of PCC worsened the acoustic insulation capabilities of PLA.

**Figure 9:**
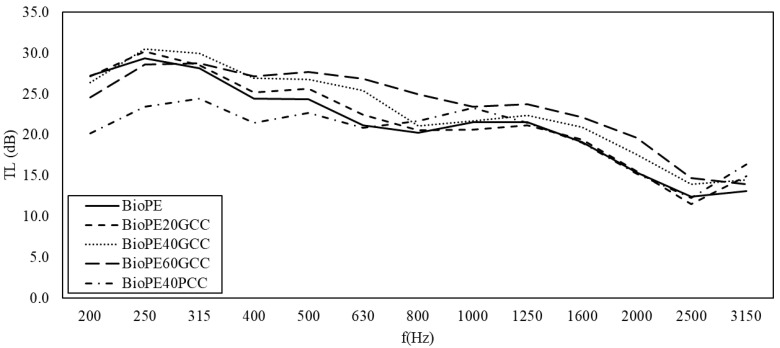
Transmission loss versus the frequency measured in the tested BioPE-based composite materials.

Figure 9 shows the transmission loss versus the frequency measured in the tested BioPE-based composite materials. Regarding BioPE, the use of GCC increased the acoustic insulation of the matrix. As in the case of PLA, the use of PCC had the opposite effect. BioPE20GCC only slightly increased the acoustic insulation of the matrix. As the percentage of GCC was increased, the acoustic insulation of the materials increased, particularly for low frequencies. Thus, in the case of BioPE, 20, 40, and 60% percentages of GCC enhanced the acoustic insulation of BioPE.

In all the cases, the transmission loss tended to decrease for frequencies above 1000 Hz. In the case of PLA40PCC, this occurred above 1600 Hz, after which the decrease was more abrupt. Gypsum boards of 12 mm showed a steady transmission loss of 30 dB up to frequencies of 2000 Hz, at which a noticeable decrease in this property was seen [27]. Polypropylene-based composites showed decreases in TL from 1280 Hz for orange tree pruning-filled materials and from 1250 Hz for wood fibers [26,27]. The response of the materials at mid-ranges was similar for the BioPE-based materials, with an advantage for the BioPE60GCC for all the frequencies. PLA-based materials showed similar responses, but the slope of the curves against frequencies was less than that of BioPE-based composites, and PLA40GCC was the best material at mid-range. At high frequencies, PLA20GCC showed better insulation. This indicates the possibility of developing mixed panels to adapt the increase in the transmission loss to the building solutions. Polyolefin filled with lignocellulosic fibers recorded better TL for frequencies from 250 to 500 Hz, with steady values around 31 dB, but decreased to 28 dB for higher frequencies [26,27]. Lignocellulosic fiber-filled polyolefin materials showed a clear link between TL and the percentage of filler. In the case of CaCO_3_, the link was not clear, and the morphology of the filler appears to play a role in the TL properties of the material, with GCC being more suitable than PCC. FE-SEM micrographs of the fillers (Figure 6) show that GCC has a softer surface than PCC, thus facilitating the total wetting of its surface without leaving air gaps. However, as revealed in Figure 8, the resultant interface between fillers and the selected matrices was apparently weak, which may induce the generation of such air gaps.

Many models used to describe the airborne insulation are based on the coupling effect between the acoustic impedances of the layers of a composite to obtain the global isolation of all the layers. The result is the index of sound reduction (R) or the transmission loss (TL) [67,68], which can be expressed as a function of the frequency or as a global value [69,70,71,72]. The numerical methods that allow the values to be obtained require an integration limit angle [73]. The influence of the limit angle on the precision of the computed values is an issue of current interest.

**Figure 10:**
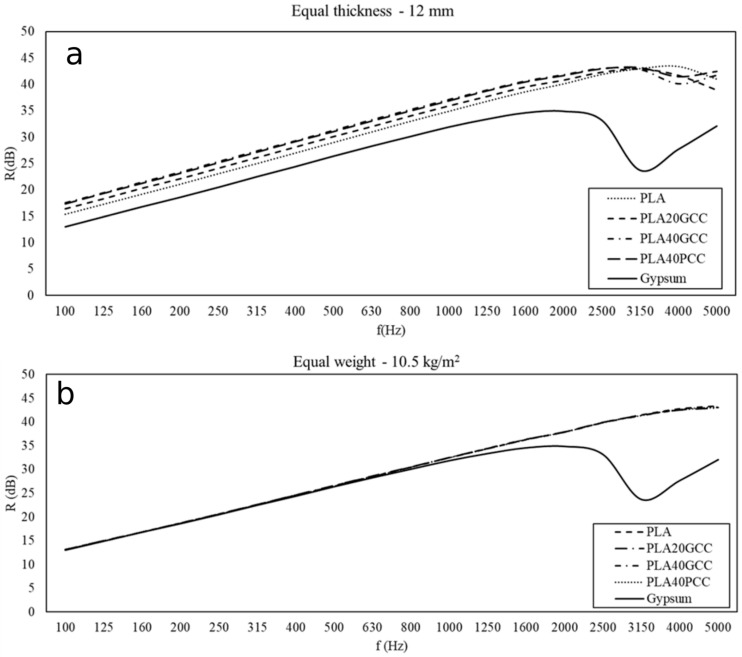
Equal thickness (**a**) and equal weight (**b**).

Figure 10a,b shows the value of the index of sound reduction that is the result of the numeric simulation for the PLA composite materials, and compared with that of a lightweight common insulation material (laminated gypsum boards). Two different simulations were undertaken, one supposing a constant thickness for all the samples (Figure 10a), and the other presuming that the mass of all the samples was the same (Figure 10b). The thickness was presumed to be 12 mm and the mass to be 10.5 kg/m^2^.

**Figure 11:**
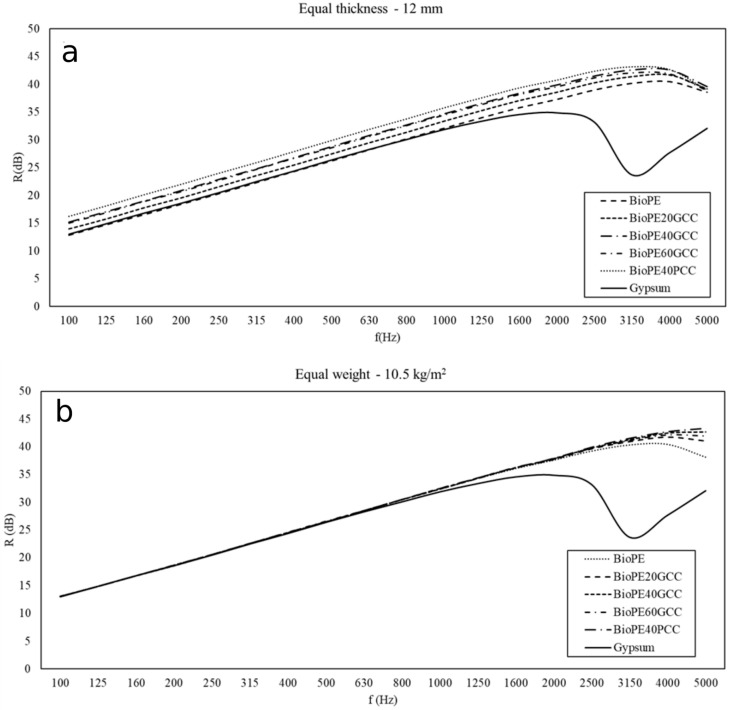
Equal thickness (**a**) and equal weight (**b**).

Figure 11a,b shows the value of the index of sound reduction that is the result of the numeric simulation for the BioPE composite materials, and compared with that of a lightweight common insulation material (laminated gypsum boards). Two different simulations were undertaken, one supposing a constant thickness for all the samples (Figure 11a), and the other presuming that the mass of all the samples was the same (Figure 11b). The thickness was presumed to be 12 mm and the mass to be 10.5 kg/m^2^.

**Figure 12:**
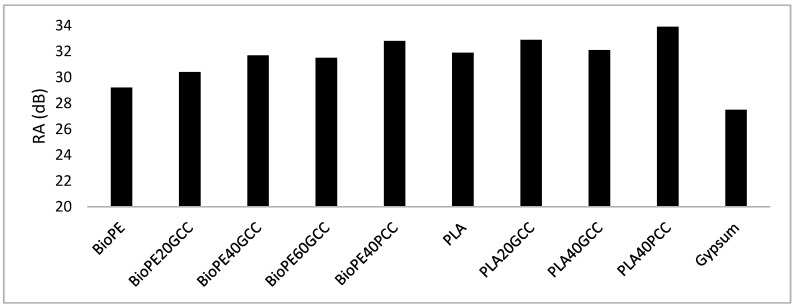
Weighted airborne sound insulation values for the different materials if all had the same 12 mm thickness.

The values for the transmission loss or insulation shown in Figure 11 and Figure 12 were obtained under diffuse field conditions. The numerical methods that allow the values to be obtained require an integration limit angle. The influence of the limit angle on the precision of the computed values is an issue of current interest. A 78° limit angle was used to obtain the values shown in Figure 11 and Figure 12. It was found that only the gypsum showed a sharp decrease in the insulation property between 2500 and 3000 Hz. All the PLA and BioPE materials have increasing acoustic insulation with increasing frequency.

Figure 11a shows that all PLA-based materials had higher acoustic insulation properties than gypsum. PLA40GCC showed the highest values. Figure 11b shows that all PLA-based materials correct the decrease in the acoustic insulation of gypsum. In the same manner, Figure 11b shows that all BioPE-based materials correct the decrease in the acoustic insulation of gypsum.

The results obtained for the insulation were similar to those obtained for TL, but it is more difficult to conclude which material offers the best insulation. When an equal weight is proposed, all the composites show similar insulations up to 3150 Hz, regardless of the matrix. The slope of the curve coincides with that of gypsum, both in the case of PLA- and BioPE-based composites. When an equal thickness hypothesis was used as a model, some materials showed higher insulation properties than others, and the differences were in the range of 2 to 4 dB. The results were similar to those of TL. In the case of lignocellulosic-reinforced polyolefin, these materials, under the same thickness hypothesis, showed better results when the percentage of reinforcement increased [26]. Nonetheless, these materials showed a decrease in insulation for frequencies higher than 2000 Hz. In this case, GCC- and PCC-filled composites showed better soundproofing properties than lignocellulosic-filled polyolefin.

**Figure 13:**
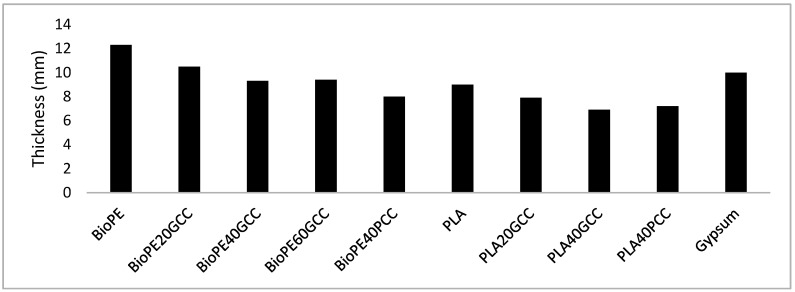
Weighted airborne sound insulation values for the different materials if all had the same weight.

The weighted airborne sound insulation values are compared in Figure 13, with the same thickness (12 mm). The maximum value was found for PLA40GCC. This material showed 6 dB more insulation than gypsum, meaning its acoustic insulation is four times higher than that of gypsum (3 dB is equivalent to double the acoustic insulation). All materials showed better airborne sound insulation than gypsum, similar to that of lignocellulosic fiber-reinforced polyolefin [26]. Nevertheless, lignocellulosic-reinforced composites showed 30 to 29 dB values, which were lower than those of CaCO_3_-based materials. In this case, particulate-filled materials offered better insulation than fiber-filled materials. By comparison, olive stone-filled polyolefin returned airborne insulation of around 26.5 dB, which is worse than that of gypsum. Thus, the aspect ratio of the filler can be concluded to be the cause of the increased or decreased airborne insulation.

Figure 13 compares the property for materials with the same weight (10.5 kg/m^2^).

All the materials, with the exception of gypsum, yielded weighted airborne sound insulation of 29.5 dB. The gypsum yielded 27.5 dB; thus, all resulted in a 2 dB increment relative to gypsum. This produced an improvement in the thickness, in general, with the most efficient being the PLA40GCC material.

In the case of the same weight hypothesis, the density of the materials played a relevant role. As a result of the density of CaCO_3_ and the matrices, materials with high CaCO_3_ contents are noticeably denser than the matrices or materials with lower filler contents. Thus, denser materials are thinner, and their soundproofing capabilities are poorer. This has implications when light weight is of importance, because gypsum will offer the best solution. Nevertheless, the response of the composites at high frequencies should be taken into account.

## 4. Conclusions

PCC exhibited a more regular diameter distribution than GCC, although it presented a crystalline structure with a sharp surface, which may limit the wettability of PCC by the matrix. By comparison, GCC was found to contain a larger number of smaller particles. However, no significant differences were found between the synthetic and the natural filler, which resulted in imperceptible differences in their mechanical performance and sound insulation. As expected, the incorporation of either GCC or PCC into PLA weakened the mechanical properties of the obtained materials, leading to a reduction in tensile strength, modulus, and elongation at break. In the case of the PLA20GCC materials, the tensile strength was 37% lower than that of the matrix, but Young’s modulus was 32% higher. However, the tensile strength of BioPE was not significantly decreased with the inclusion of either PCC or GCC, which is an advantage. By comparison, Young’s modulus of BioPE-based composites increased noticeably, and was 67% higher than that of the matrix for a BioPE60GCC composite. Flexural strength of the PLA-based materials decreased with filler contents, decreasing 69% relative to that of the matrix for a PLA40GCC composite. The flexural modulus for the same material increased by 22%. In the case of BioPE-based materials, flexural strength increased with the presence of the filler, by up to 43% for BioPE60GCC.

Impact strength changed noticeably with the presence of fillers. In the case of PLA-based materials, notched specimens increased from 24.5 kJ/m^2^ (PLA matrix) to 2.73 kJ/m^2^ (PLA40GCC). In the case of notched specimens, the values increased from 2.9 kJ/m^2^ to a value lower than 0.5 J. The BioPE-based material under the unnotched test increased from a value higher than 5.5 J for the matrix, to 49.61 kJ/m_2_ for BioPE60GCC composite, reducing the impact strength of the matrix, but remaining higher than that of PLA-based composites. The reduction in the notched impact strength for the same materials was lower, decreasing from 6.66 kJ/m^2^ to 5.17 kJ/m^2^.

In all cases, the incorporation of mineral fillers enhanced the acoustic insulation of PLA and BioPE, and resulted in a higher performance than gypsum, particularly for frequencies higher than 2000 Hz. PLA- and BioPE-based materials showed similar soundproofing enhancements compared to gypsum boards. PLA40GCC obtained 6 dB more airborne insulation than that of gypsum, thus achieving a four-fold increase in the acoustic insulation.

This provides an opportunity for bio-based thermoplastics filled with calcium carbonate, which can be easily processed and have a competitive production cost, particularly at high filler contents. PLA-based composites, although reported to be biodegradable, do not offer good mechanical performance due to their brittleness, which may be a limiting factor when considering the use of these materials for construction purposes.

## Figures and Tables

**Table 1:** Density of the obtained composites.

Specimen	Density (kg/m^3^)
PLA	1165
PLA20GCC	1316
PLA40GCC	1526
PLA40PCC	1477
BioPE	846
BioPE20GCC	979
BioPE40GCC	1137
BioPE60GCC	1293
BioPE40PCC	1117

**Table 2:** Color coordinates of the obtained PLA and BioPE composites.

PLA	PLA20GCC	PLA40GCC	PLA40PCC	
H: 69	H: 90	H: 96	H: 82	
S: 15	S: 10	S: 8	S: 9	
L: 75	L: 76	L: 75	L: 77	
**BioPE**	**BioPE20GCC**	**BioPE40GCC**	**BioPE60GCC**	**BioPE40PCC**
H: 190	H: 88	H: 90	H: 96	H: 69
S: 16	S: 9	S: 9	S: 8	S: 10
L: 78	L: 72	L: 73	L: 74	L: 73

**Table 3:** Tensile properties of PLA and BioPE composites filled with CaCO_3_.

	σ_t_ (MPa)	ε_tM_ (%)	ε_t_ (%)	E_t_ (GPa)
PLA	62.58 ± 0.83 ^e^	3.284 ± 0.073 ^b^	4.224 ± 0.033 ^b^	3.821 ± 0.110 ^e^
PLA20GCC	45.48 ± 0.99 ^d^	1.871 ± 0.106 ^a^	1.893 ± 0.127 ^a^	5.042 ± 0.070 ^f^
BioPE	18.05 ± 0.159 ^b^	12.180 ± 525 ^e^	>100mm ^e^	1.060 ± 0.028 ^a^
BioPE20GCC	16.07 ± 0.16 ^a^	7.595 ± 0.406 ^d^	23.430 ± 1.409 ^f^	1.265 ± 0.058 ^b^
BioPE40GCC	17.55 ± 0.51 ^b^	6.174 ± 0.211 ^c^	10.830 ± 0.204 ^d^	1.530 ± 0.006 ^c^
BioPE40PCC	17.97 ± 0.26 ^b,c^	6.510 ± 0.430 ^c^	8.434 ± 1.195 ^c^	1.499 ± 0.083 ^c^
BioPE60GCC	19.15 ± 0.15 ^c^	6.214 ± 0.181 ^c^	6.474 ± 0.211 ^c^	1.785 ± 0.038 ^d^

Different letters a, b, c, d, e, and f represent the statistical difference (ANOVA, *p* < 0.05) between the properties of the materials.

**Table 4:** Flexural properties of PLA and BioPE composites filled with CaCO_3_.

	σ_f_ (MPa)	D (mm)	ε_f_ (%)	E_f_ (GPa)
PLA	114.10 ± 1.21 ^f^	6.975 ± 0.073 ^c^	4.69 ± 0.05 ^c^	3.987 ± 0.0153 ^d^
PLA20GCC	68.13 ± 1.18 ^e^	4.167 ± 0.106 ^b^	2.80 ± 0.07 ^b^	3.848 ± 0.0048 ^d^
PLA40GCC	20.41 ± 0.74 ^a^	0.646 ± 0.093 ^a^	0.43 ± 0.06 ^a^	4.843 ± 0.0063 ^e^
BioPE	21.25 ± 0.34 ^a^	9.790 ± 0.054 ^f^	6.58 ± 0.04 ^f^	0.839 ± 0.0025 ^a^
BioPE20GCC	23.84 ± 0.49 ^b^	8.938 ± 0.031 ^e^	6.01 ± 0.02 ^e^	0.965 ± 0.0020 ^a,b^
BioPE40GCC	27.60 ± 0.27 ^c^	8.996 ± 0.091 ^e^	6.05 ± 0.06 ^e^	1.257 ± 0.0002 ^b,c^
BioPE40PCC	28.33 ± 0.37 ^c,d^	8.692 ± 0.045 ^d^	5.84 ± 0.03 ^d^	1.341 ± 0.0014 ^b,c^
BioPE60GCC	30.52 ± 0.60 ^d^	8.912 ± 0.040 ^d,e^	5.99 ± 0.03 ^d,e^	1.633 ± 0.0123 ^c^

Different letters a, b, c, d, e, and f represent the statistical difference (ANOVA, *p* < 0.05) between the properties of the materials.

**Table 5:** Impact strength of PLA and BioPE composites filled with CaCO_3_.

	Charpy Unnotched (kJ/m^2^)	Charpy Notched (kJ/m^2^)	Izod (J/m)
PLA	24.46 ± 1.10 ^d^	2.90 ± 0.16 ^b^	34.51 ± 0.86 ^b,c^
PLA20GCC	16.48 ± 0.76 ^c^	2.64 ± 0.21 ^b^	27.99 ± 1.12 ^b^
PLA40GCC	2.73 ± 0.85 ^b^	<0.5J ^a^	2.44 ± 1.99 ^a^
BioPE	>5.5J ^a^	6.66 ± 0.73 ^f^	52.67 ± 2.72 ^e^
BioPE20GCC	>5.5J ^a^	5.94 ± 0.08 ^e^	49.38 ± 5.58 ^e^
BioPE40GCC	43.65 ± 0.78 ^f^	3.63 ± 0.06 ^c^	40.40 ± 3.86 ^c,d^
BioPE40PCC	35.76 ± 1.13 ^e^	3.67 ± 0.08 ^c^	38.01 ± 3.06 ^c,d^
BioPE60GCC	49.61 ± 2.24 ^g^	5.17 ± 0.23 ^d^	45.96 ± 8.28 ^d,e^

Different letters a, b, c, d, e, f, and g represent the statistical difference (ANOVA, *p* < 0.05) between the properties of the materials.
